# Diagnostic accuracy of deep learning using ultra-widefield fundus imaging for retinal detachment: a systematic review and meta-analysis

**DOI:** 10.1186/s12886-025-04605-8

**Published:** 2026-01-03

**Authors:** Chiaki Kawamoto, Yuki Mizuki, Nobuyuki Horita, Yuichi Kuramochi, Shun Kanasashi, Tatsukata Kawagoe, Nobuhisa Mizuki

**Affiliations:** 1 Department of Ophthalmology and Visual Science, Yokohama City University Graduate School of Medicine, 3-9 Fukuura, Kanazawa-ku, Yokohama, 236-0004 Japan; 2 Department of Ophthalmology, Yokohama Minami Kyosai Hospital, Yokohama, Japan; 3 Chemotherapy Center, Yokohama City University Hospital, Yokohama, Japan; 4 Department of Ophthalmology, Fujisawa City Hospital, Fujisawa, Japan; 5 Department of Ophthalmology, Dokkyo Medical University, Tochigi, Japan

**Keywords:** Retinal detachment, Deep learning, Ultra-widefield imaging, Convolutional neural networks, Diagnostic accuracy, Meta-analysis

## Abstract

**Background:**

Retinal detachment (RD) requires prompt detection to prevent vision loss. Ultra-widefield (UWF) imaging captures the peripheral retina, and deep learning (DL) may enable automated RD detection. We aimed to systematically review and meta-analyze the diagnostic accuracy of DL applied to UWF images for detecting RD.

**Methods:**

We systematically searched PubMed, Web of Science, and reference lists (last search 22 May 2025) for diagnostic-accuracy studies evaluating DL models for retinal detachment on UWF images with extractable 2 × 2 data. Two reviewers independently selected studies, extracted data, and assessed risk of bias and concerns regarding applicability using the Quality Assessment of Diagnostic Accuracy Studies-2 (QUADAS-2). Sensitivity, specificity, and area under the curve (AUC) were pooled using random-effects models, with subgroup analyses by dataset origin (internal vs. external) and retinal detachment spectrum.

**Results:**

We included 11 studies (2017–2024) using UWF imaging and DL, with test set sizes ranging from 89 to 6,222 images. The pooled sensitivity and specificity were 0.95 (95% CI, 0.94–0.96) and 0.99 (95% CI, 0.99–0.99); the AUC of the summary receiver operating characteristic (SROC) = 0.9962. Heterogeneity was high (I² = 92% for sensitivity; 90% for specificity). In subgroup analyses, external evaluations showed higher sensitivity than internal ones (0.97 vs. 0.92), with similarly high specificity (both ≈ 0.99). Heterogeneity remained substantial within subgroups. QUADAS-2 indicated a low risk of bias in most domains, with unclear index test risk common due to non-prespecified thresholds.

**Conclusions:**

DL applied to UWF imaging shows high diagnostic accuracy for RD, with pooled sensitivity and specificity of 0.95 and 0.99, respectively, and an AUC of 0.9962. However, the evidence is limited by substantial heterogeneity, inconsistent index-test reporting, and variation in case spectrum and sample size, which may constrain generalizability. Overall, these findings suggest that DL combined with UWF imaging is likely to serve as a valuable adjunctive tool for RD detection and triage, particularly in settings where rapid, wide-field assessment is needed.

**Registration:**

UMIN-CTR UMIN000057903; PROSPERO CRD420251058209.

**Supplementary Information:**

The online version contains supplementary material available at 10.1186/s12886-025-04605-8.

## Introduction

Retinal detachment (RD) is an ophthalmic emergency in which the neurosensory retina separates from the retinal pigment epithelium; most cases are rhegmatogenous retinal detachment (RRD) [1, 2]. The incidence of RD is estimated at around 1 in 10,000 people annually and appears to be increasing with aging populations, rising myopia, and more frequent cataract surgeries [2–4]. Visual prognosis depends strongly on whether the macula is still attached, so early diagnosis and intervention are critical [2]. Indirect ophthalmoscopy and B-scan ultrasonography are standard for RD detection but require pupil dilation, technical expertise, and patient cooperation, and their performance is examiner dependent [5, 6]. In contrast, ultra-widefield (UWF) imaging provides a noncontact, wide-field view of up to 200 degrees in a single shot, with high reproducibility and efficiency, and is now widely used for retinal screening and documentation [7, 8].

Recent advances in deep learning (DL), especially those based on convolutional neural networks (CNNs), have revolutionized image-based diagnostics in ophthalmology. DL algorithms applied to fundus photograph and optical coherence tomography (OCT) have demonstrated high diagnostic accuracy in identifying diseases such as diabetic retinopathy, age-related macular degeneration, and glaucoma [9, 10].

DL can automatically extract features through its multi-layered architecture, enhancing diagnostic efficiency, standardization, and reproducibility. This supplements the limitations of human interpretation and reduces the burden on patients and healthcare providers. DL is compatible with telemedicine and is gaining attention as an automated screening and triage tool in primary care, emergency, and tele-ophthalmology settings, where it can support, rather than replace, specialist assessment for conditions such as RD [10–12].

Recent studies have proposed DL algorithms utilizing UWF images for RD diagnosis. These include DL models that automatically detect RRD and typical peripheral retinal findings in UWF images. However, there is heterogeneity across studies in terms of disease definitions, algorithm architectures, and evaluation metrics, including sensitivity, specificity, and area under the curve (AUC), which hinders consistent comparisons and synthesis of diagnostic performance [13, 14].

This systematic review and meta-analysis aimed to quantify the diagnostic accuracy (sensitivity, specificity, and AUC) of DL algorithms applied to UWF fundus images for detecting RD in patients undergoing UWF imaging, using comprehensive ophthalmic examination as the reference standard. By synthesizing the current evidence, this study may provide support for earlier diagnosis, improved visual outcomes, and more efficient healthcare delivery.

## Methods

### Protocol and registration

This protocol has been registered with both the University Hospital Medical Information Network Clinical Trials Registry (UMIN-CTR; UMIN000057903; registration date: May 19, 2025; https://center6.umin.ac.jp/cgi-open-bin/ctr/ctr_view.cgi?recptno=R000066189) and the International Prospective Register of Systematic Reviews (PROSPERO; CRD420251058209; https://www.crd.york.ac.uk/PROSPERO/view/CRD420251058209). The study follows PRISMA-DTA and Cochrane Handbook recommendations [15, 16].

#### Amendments

Minor deviations from the registered protocol were made. Specifically, the final analysis used Meta-DiSc (version 1.4) with univariate random-effects models (DerSimonian–Laird) and a symmetric summary receiver operating characteristic (SROC) curve (Moses–Littenberg) instead of the prespecified bivariate model, because the small number of eligible studies made the bivariate model difficult to fit reliably. In addition, subgroup analyses were conducted by dataset origin (internal vs. external) and disease subtype (RRD-only or mixed-type RD cohorts [i.e., cohorts in which the RD group included RRD and other RD subtypes]), rather than by model type or image quality as initially planned, as these factors were judged a priori to be the most clinically relevant sources of heterogeneity for DL performance. Cohorts restricted to recurrent/postoperative RD were treated separately as an extreme-spectrum population. These amendments have been documented, and the PROSPERO record was updated accordingly.

### Eligibility criteria

We included human diagnostic-accuracy clinical studies (case-control, cross-sectional, prospective, or retrospective) that evaluated DL algorithms applied to UWF images for the detection of RD in clinical settings and reported sufficient information to reconstruct diagnostic 2 × 2 tables (e.g. explicit TP, FP, FN, and TN counts or sensitivity and specificity together with the numbers of RD and non-RD images in the test set). We recorded the target-condition spectrum reported in each study (RRD-only and mixed-type RD cohorts); cohorts restricted to recurrent/postoperative RD were treated as an extreme-spectrum population in sensitivity analyses. Studies were eligible if they enrolled patients with suspected or confirmed RD and allowed derivation of a complete 2 × 2 contingency table at the image or eye level, using clinical diagnosis by an ophthalmologist based on dilated fundus examination as the reference standard. In accordance with the registered PROSPERO protocol (CRD420251058209), we considered peer-reviewed full-text articles and eligible conference abstracts from database inception to 22 May 2025, with no restrictions on publication date, language, or patient age, provided that adequate methodological information and reconstructable 2 × 2 data were available. We excluded non-human studies, non-UWF imaging, studies evaluating only non–DL methods, simulation-only studies, case reports, reviews, editorials, letters without original data, and reports that did not allow reconstruction of complete 2 × 2 data (e.g. reporting only sensitivity or only specificity without denominators or cell counts), because complete 2 × 2 data were required for pooled diagnostic accuracy estimates.

### Information sources and search

Two independent reviewers (CK and YM) systematically searched the PubMed and Web of Science databases from inception to 22 May 2025. They also screened the reference lists of included articles and relevant clinical guidelines to identify additional studies, and manual reference screening was completed on the same date. We did not search trial registries or contact study authors for additional unpublished data. The full electronic search strategies for each database, including all limits and filters used, are provided in Supplementary Material.

### Study selection

Two reviewers (CK and YM) independently screened the titles and abstracts of all retrieved records and assessed the full texts of potentially eligible studies against the predefined eligibility criteria. Disagreements were resolved through discussion, and no dedicated screening software or other automation tools were used; all screening was performed manually. Duplicate datasets were identified and excluded as necessary. Studies that met the eligibility criteria and provided extractable 2 × 2 data were included in the systematic review and meta-analysis of diagnostic accuracy outcomes (sensitivity, specificity, AUC) after confirming comparability in the definitions of retinal detachment across studies.

### Data collection process and definitions for data extraction

Two reviewers (CK and YM) independently and in duplicate extracted study characteristics, including the first author’s name, year of publication, study country, number of images in the test dataset, retinal detachment type, diagnostic reference standard and examiner expertise, ultra-widefield camera model, DL architecture, reported AUC, and information related to the Quality Assessment of Diagnostic Accuracy Studies-2 (QUADAS-2) [17]. Data were recorded in a standardized Microsoft Excel spreadsheet (Microsoft Corp., Redmond, WA, USA) for collation and checking. For data extraction, we defined the target condition as RD, the index test as any DL model applied to UWF fundus images, the reference standard as ophthalmologist diagnosis based on dilated fundus examination, and study designs as case-control, cross-sectional, prospective, or retrospective clinical studies; the clinical setting was recorded when reported. No contact with study investigators was required, and no automation tools were used in the data collection process. Missing or unclear information was noted as “Unclear,” with no post hoc assumptions made beyond reconstructing 2 × 2 tables from the reported percentages and denominators.

Apart from reconstructing 2 × 2 tables as described above, no additional data transformations were performed. Sensitivity and specificity were calculated from reported TP, FP, FN, and TN counts; when these cell counts were incomplete, we reconstructed the 2 × 2 tables from the available combination of reported accuracy measures (e.g. sensitivity, specificity) and the numbers of RD and non-RD images in the test set.

### Risk of bias and concerns regarding applicability

Two reviewers (CK and YM) independently assessed the risk of bias and concerns regarding applicability for each included study using the QUADAS-2 [17], which is specifically designed for diagnostic accuracy reviews. QUADAS-2 assesses risk of bias across four domains (patient selection, index test, reference standard, and flow and timing) and concerns regarding applicability across three domains (patient selection, index test, and reference standard). We judged risk of bias across the four domains and concerns regarding applicability across the three applicable domains as low, high, or unclear according to prespecified criteria. No automation tools were used for the judgments in this process. Disagreements were resolved through discussion until consensus was reached. We did not derive an overall quality score; instead, we reported domain-level QUADAS-2 judgments and identified studies with any domain rated as high risk of bias for sensitivity analyses. The QUADAS-2 traffic-light and summary plots were generated using the robvis visualization tool [18].

### Diagnostic accuracy measures

The primary outcomes were sensitivity, specificity, and AUC, which were calculated from the extracted true positive (TP), false positive (FP), false negative (FN), and true negative (TN) values. SROC curves were plotted accordingly.

We extracted results from test sets rather than validation sets to ensure independent evaluation. External test sets were prioritized whenever available because they better reflect generalizability. If multiple test sets were reported, we prioritized external or prospectively collected sets; otherwise, we used the primary internal set as designated by the original authors. When more than one eligible set remained, we selected the largest and most independent set. When no external evaluation was available, the internal test set was used. If a prospective evaluation was conducted, results from the prospective test set were prioritized. In all cases, when the authors explicitly designated a primary evaluation (e.g., in the abstract or main results), that result was selected.

### Synthesis of results

Heterogeneity was assessed using I². Subgroup and sensitivity analyses were conducted to explain, rather than eliminate, heterogeneity. To ensure comparability across studies, we synthesized only binary classifications of RD versus non-RD at a single author-designated decision threshold, extracting one pair of sensitivity and specificity values per study based on the primary test set (prioritizing external or prospective test sets when available, as described above); no studies reported multiple thresholds, multiple index-test readers, or indeterminate test results requiring special handling, and all reference standards were based on dilated fundus examination by ophthalmologists. Because the number of studies per synthesis was small, meta‑regression was not performed. Formal assessments of reporting/publication bias were not undertaken for subgroups; however, Deeks’ funnel asymmetry test was explored for the overall synthesis only. Certainty of evidence was not graded; instead, study-level risk of bias was evaluated with QUADAS-2.

### Meta-analysis

We calculated study-level sensitivity and specificity from 2 × 2 tables. Pooled estimates were obtained using random-effects models (DerSimonian–Laird) on the logit scale, fitted separately for sensitivity and specificity, as implemented in Meta-DiSc v1.4 (Unidad de Bioestadística Clínica, Hospital Ramón y Cajal, Madrid, Spain). A symmetric SROC curve (Moses–Littenberg method) was generated together with its AUC. When any cell count was zero, we applied a continuity correction of 0.5. Diagnostic odds ratios (DORs) were also computed under random effects. Heterogeneity was quantified using I² (0% no, > 0%–<25% minimal, 25%–<50% mild, 50%–<75% moderate, ≥ 75% strong). Statistical significance was set at *p* < 0.05. Because only 11 studies were available and several potentially relevant covariates (e.g., dataset origin, study design, disease spectrum) were correlated, we did not perform meta-regression, as such analyses would have been statistically underpowered and likely to yield unstable estimates in this setting.

In addition, for the overall synthesis we explored small-study effects using Deeks’ funnel asymmetry test (weighted regression of log DOR on 1/√ESS) in R 4.5.1, applying the same continuity correction as above; subgroup assessments were not undertaken because study numbers were small and such tests are unreliable in DTA meta-analyses.

### Additional analyses

To explain heterogeneity without over-stratification, we examined two subgroup contrasts that were clinically and methodologically most relevant to this review: (i) dataset origin (internal vs. external, the latter including independent-center and/or temporally independent test sets), and (ii) RD subtype (RRD-only cohorts vs. mixed-type RD cohorts in which the RD group included RRD and other RD subtypes). Cohorts restricted to recurrent/postoperative RD were treated separately as an extreme-spectrum population and evaluated in sensitivity analyses. Sensitivity analyses were performed by repeating the meta-analysis after excluding (1) studies at high risk of bias (any QUADAS-2 risk-of-bias domain rated high), (2) extreme-spectrum studies (population screening cohorts and cohorts restricted to recurrent/postoperative RD), and (3) both high-risk-of-bias and extreme-spectrum studies. For each stratum with ≥ 3 studies, we re-fitted the same random-effects models (DerSimonian–Laird on the logit scale for sensitivity and specificity) and derived symmetric SROC AUCs using Meta-DiSc v1.4. No further subgrouping or meta-regression was undertaken to avoid unstable estimates given the limited number of studies per stratum.

## Results

### Study selection

**Fig. 1:**
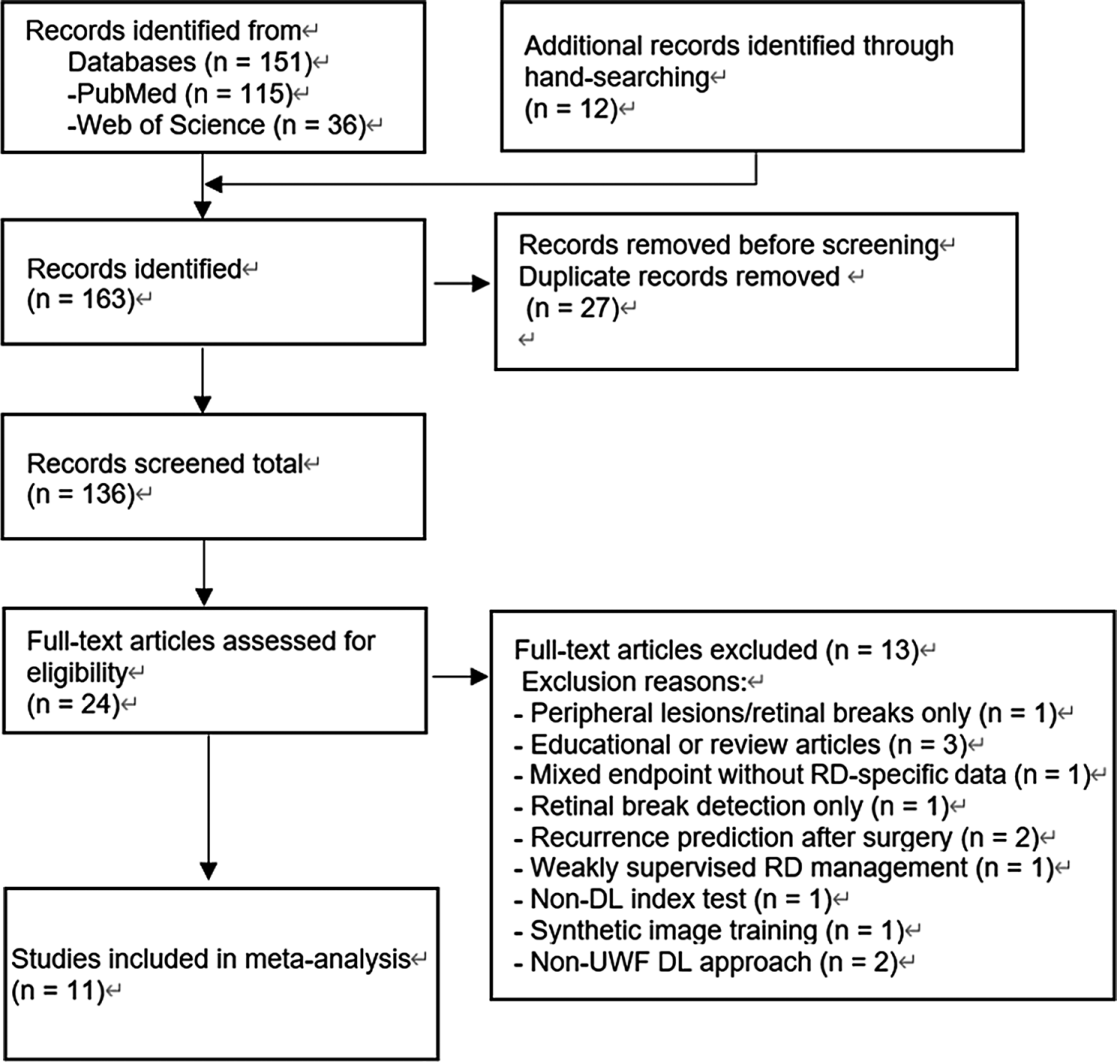
PRISMA flow diagram. A total of 151 records were identified through database searches, with 12 additional records found through hand-searching. After removing duplicates, 136 unique records underwent title and abstract screening, leaving 24 articles for full-text review. Of these, 13 were excluded for reasons outlined in Supplementary Table S1, resulting in 11 studies included in the meta-analysis. Abbreviations: PRISMA = Preferred Reporting Items for Systematic Reviews and Meta-Analyses; RD = retinal detachment; DL = deep learning

A total of 151 records were identified through electronic database searches, including 115 from PubMed and 36 from Web of Science. An additional 12 records were identified through hand-searching of references and relevant guidelines. After removing duplicates, 136 unique records remained for title and abstract screening. Of these, 112 were excluded, and 24 full-text articles were assessed for eligibility. After full-text review, 13 articles were excluded for various reasons, and 11 studies were ultimately included in the meta-analysis (Fig. 1). Details of the excluded studies that appeared eligible, along with reasons for their exclusion, are provided in Supplementary Table S1.

### Study characteristics

Eleven studies published between 2017 and 2024 were included in this meta-analysis. The studies were conducted in China (*n* = 8) [14, 19–25], Japan (*n* = 1) [26], Canada (*n* = 1) [13], and Switzerland (*n* = 1) [27]. Sample sizes ranged from 89 to 6,222 images. All studies employed ultra-widefield (UWF) fundus imaging using devices such as the Optos 200Tx (Optos), Daytona (Optos), Optos P200 (Optos), and Clarus 500 (Carl Zeiss). In some cases, multiple devices were used within a single study.

DL techniques varied across studies and included CNNs with architectures such as Inception-ResNet-V2, seResNext50, EfficientNet-B0 and B7, Inception-V3, Xception, YOLOX, and the four-hierarchical interpretable eye diseases screening system as well as ensemble and multilabel approaches. One study employed automated machine learning (AutoML), and in another, the specific DL technique was unclear. Several studies developed multilabel classifiers for detecting RD and other peripheral retinal lesions such as retinal breaks and lattice degeneration. The reference standard in most studies involved diagnosis by experienced retinal specialists, although one study did not explicitly specify diagnostic criteria. A concise overview of the included studies is presented in Table 1, and detailed model architectures and reference standards are summarized in Supplementary Table S2.

**Table 1:** Characteristics of included studies

Study	Country	Disease	Dataset type	*N* (RD proportion (%))	Study design	Clinical setting
Ohsugi 2017 [26]	Japan	RRD	Internal	831 (49.5%)	Retrospective	Single-centre hospital-based UWF screening
Li 2020 [19]	China	Mixed-type RD	External (Zhongshan)	400 (50.0%)	Retrospective	Single-centre hospital-based UWF screening
Zhang 2021 [20]	China	Mixed-type RD	Internal	189 (8.5%)	Retrospective	Single-centre hospital-based UWF screening
Zhou 2022 [21]	China	Recurrent/postoperative RD	Temporal-external (same institution)	89 (60.7%)	Prospective	Single-centre postoperative recurrent RD follow-up screening
Cao 2022 [22]	China	Mixed-type RD	External (the First Affiliated Hospital of University of Science and Technology)	3694 (14.4%)	Retrospective	Multicentre hospital-based UWF screening
Sun 2023 [23]	China	Mixed-type RD	External (Wuhan Optics Valley)	979 (13.8%)	Retrospective	Single-centre hospital-based UWF screening
Antaki 2023 [13]	Canada	Mixed-type RD	Internal	213 (41.3%)	Retrospective	Multicentre hospital-based UWF screening (Public database)
Tang 2023 [24]	China	RRD	Internal	881 (16.6%)	Retrospective	Single-centre hospital-based UWF screening
Cui 2023 [25]	China	Mixed-type RD	External (rural screening)	6222 (0.1%)	Prospective	Rural population UWF screening
Wang 2023 [14]	China	RRD	External (West China Hosp.)	600 (10.8%)	Retrospective	Single-centre hospital-based UWF screening
Christ 2024 [27]	Switzerland	Mixed-type RD	Internal	481 (36.6%)	Retrospective	Single-centre hospital-based UWF screening

Summary of studies included in the meta-analysis evaluating the diagnostic performance of deep learning (DL) algorithms using ultra-widefield (UWF) fundus imaging for retinal detachment (RD). The table presents study origin, target-condition spectrum (RRD-only; mixed-type RD, defined as cohorts in which the RD group included RRD and other RD subtypes; and recurrent/postoperative RD, treated as an extreme-spectrum population), dataset characteristics, the number and proportion of RD and non-RD images (class balance), together with study design and clinical setting

Abbreviations:

RD = retinal detachment; RRD = rhegmatogenous retinal detachment; UWF = ultra-widefield

### Risk of bias and concerns regarding applicability

Risk of bias and concerns regarding applicability were assessed with QUADAS-2 [17]. For patient selection (risk of bias), four studies were judged high risk owing to selective or enriched sampling (Ohsugi 2017 [26], Cao 2022 [22], Antaki 2023 [13], Cui 2023 [25]); the remainder were low risk. For the index test (risk of bias), nine studies were rated unclear because prespecified thresholds and/or blinding were insufficiently described (Ohsugi [26], Li [19], Zhang [20], Zhou [21], Sun [23], Tang [24], Cui [25], Wang [14], Christ [27]); Cao [22] and Antaki [13] were low risk. For the reference standard (risk of bias), four studies were unclear due to limited reporting of diagnostic criteria or adjudication (Ohsugi [26], Li [19], Antaki [13], Cui [25]); others were low risk. Flow and timing were low risk in most studies, with unclear ratings for Ohsugi [26] and Li [19] and high ratings for Cao [22] and Antaki [13].

**Fig. 2:**
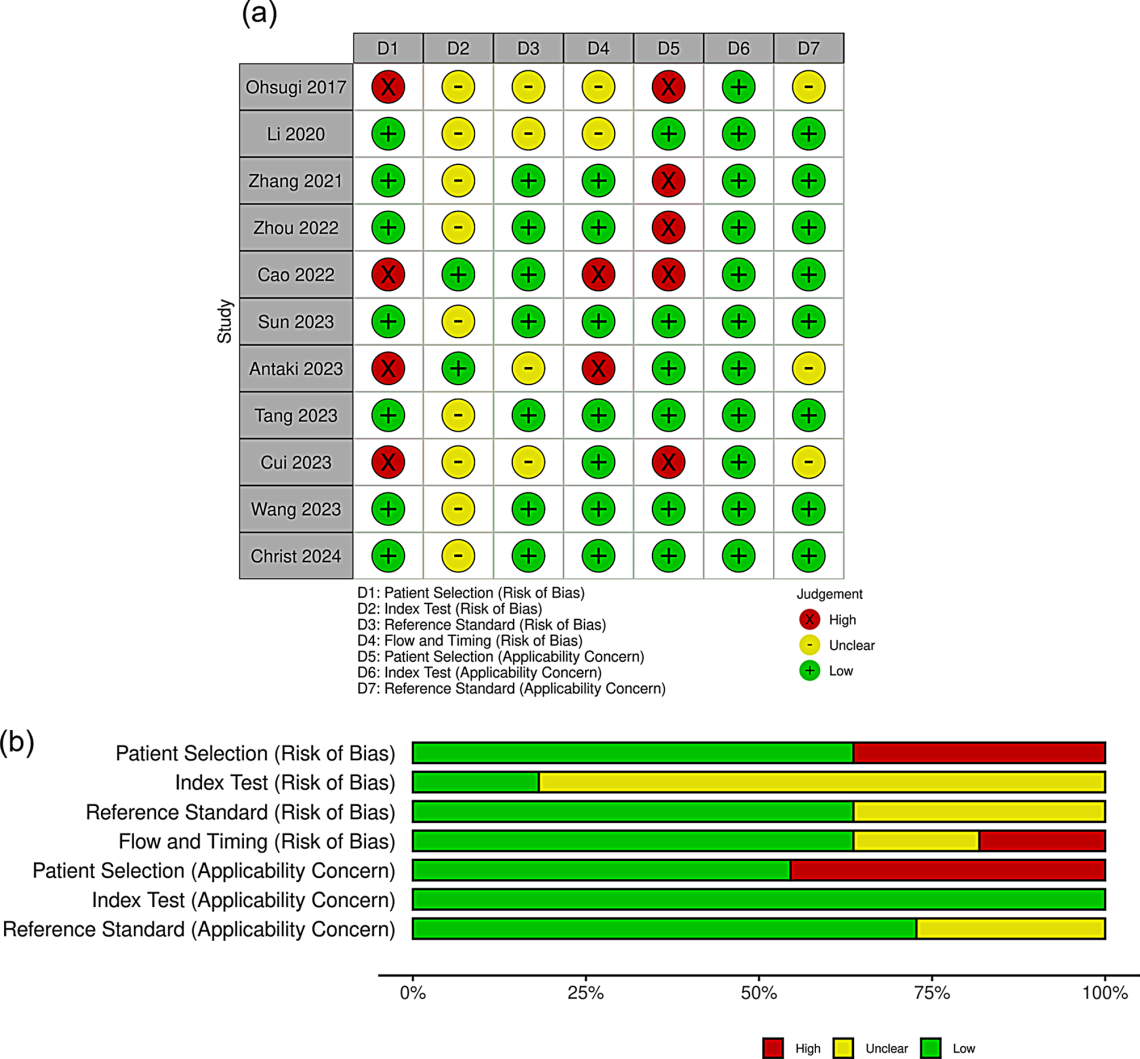
Risk of bias and applicability concerns for included studies assessed with QUADAS-2. (**a**) Traffic-light plot showing study-level judgments across four risk-of-bias domains and three applicability concern domains. Green indicates low risk or concern, yellow indicates unclear risk or concern, red indicates high risk or concern. D1, Patient Selection (risk of bias); D2, Index Test (risk of bias); D3, Reference Standard (risk of bias); D4, Flow and Timing (risk of bias); D5, Patient Selection (applicability concern); D6, Index Test (applicability concern); D7, Reference Standard (applicability concern). (**b**) Summary bar plots showing the proportion of studies rated at each level of risk of bias or applicability concern. Green indicates low risk or concern, yellow indicates unclear risk or concern, red indicates high risk or concern. Abbreviations: QUADAS-2, Quality Assessment of Diagnostic Accuracy Studies-2

Regarding concerns regarding applicability, patient selection concerns were high in Ohsugi [26], Zhang [20], Zhou [21], Cao [22], and Cui [25], reflecting spectrum differences from routine clinical screening, and low in the remaining studies. Concerns regarding applicability for the index test were low in all studies. Concerns regarding applicability for the reference standard were unclear in Ohsugi [26], Antaki [13], and Cui [25], and low otherwise. These QUADAS-2 risk-of-bias and concerns regarding applicability assessments are summarized in Fig. 2.

## Results of individual studies

**Fig. 3:**
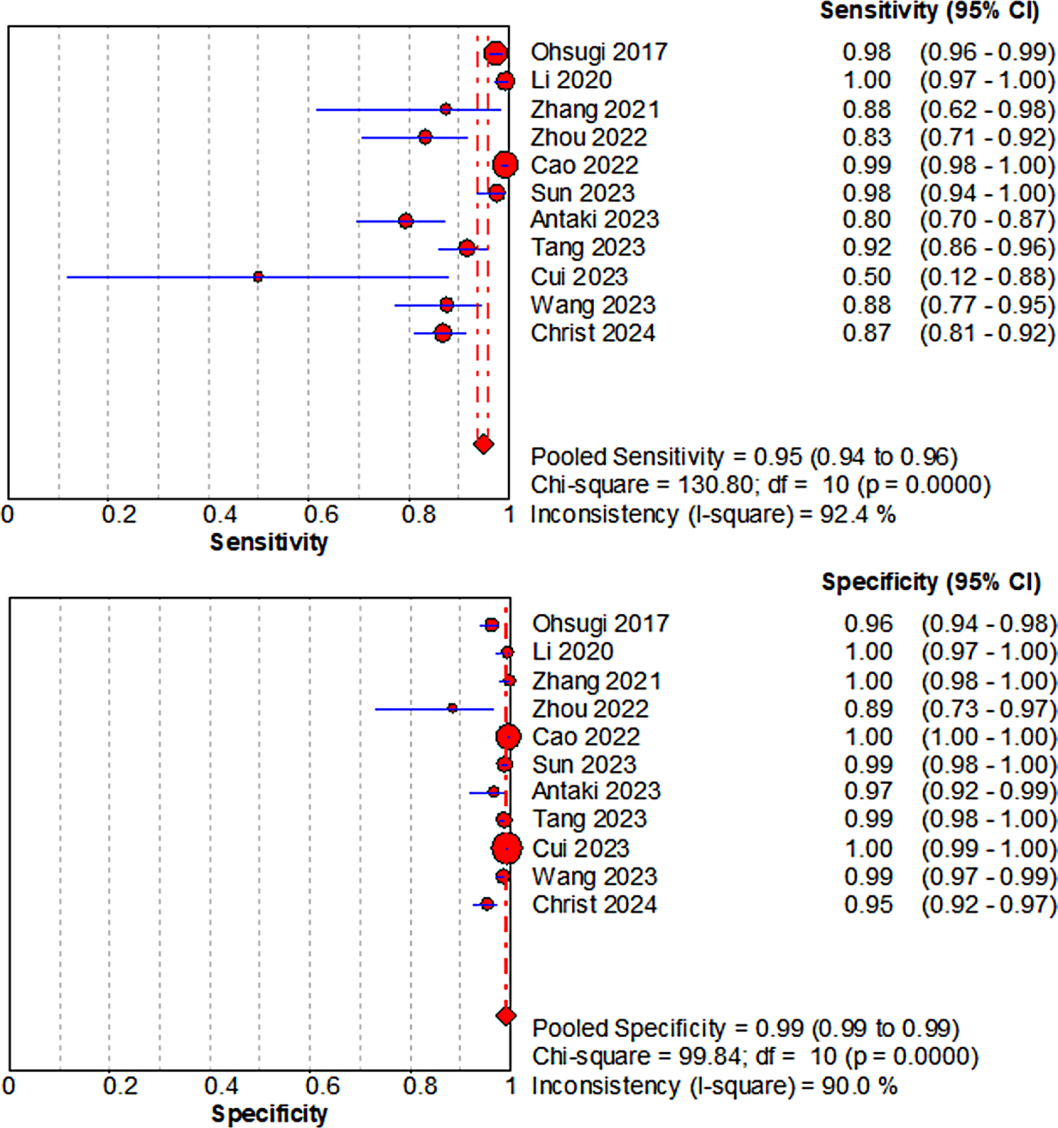
Forest plots of sensitivity and specificity for deep learning diagnosis of retinal detachment using ultra-widefield imaging. Forest plots illustrating individual study estimates and pooled estimates of sensitivity (left) and specificity (right) for deep learning-based detection of retinal detachment using ultra-widefield fundus images. The pooled sensitivity was 0.95 (95% CI: 0.94–0.96) with significant heterogeneity (I² = 92.4%). The pooled specificity was 0.99 (95% CI: 0.99–0.99), also showing substantial heterogeneity (I² = 90.0%). Abbreviations: CI = confidence interval

Study-level 2 × 2 data and accuracy estimates for the primary test set in each study were summarized in Table 2. Across the 11 test sets, sensitivity ranged from 0.50 to 1.00 and specificity from 0.89 to 1.00. In most studies, sensitivity was ≥ 0.88 and specificity was ≥ 0.95, whereas lower sensitivity was observed in the recurrent RD cohort of Zhou et al. and in the population-based screening study by Cui et al. AUC values, where reported, ranged from 0.91 to 1.00. Individual study estimates with 95% confidence intervals were displayed in the forest plots for sensitivity and specificity (Fig. 3).

**Table 2:** Diagnostic performance of deep learning models in individual studies

Study	RD subtype	Test set (RD/non-RD)	TP	FN	FP	TN	Sensitivity	Specificity	AUC
Ohsugi 2017 [26]	RRD	411/420	401	10	15	405	0.98	0.96	0.988
Li 2020 [19]	Mixed-type RD	200/200	199	1	1	199	1.00	1.00	1.000
Zhang 2021 [20]	Mixed-type RD	16/173	14	2	0	173	0.88	1.00	1.000
Zhou 2022 [21]	Recurrent/postoperative RD	54/35	45	9	4	31	0.83	0.89	0.930
Cao 2022 [22]	Mixed-type RD	532/3162	529	3	7	3155	0.99	1	NR
Sun 2023 [23]	Mixed-type RD	135/844	132	3	8	836	0.98	0.99	0.998
Antaki 2023 [13]	Mixed-type RD	88/125	70	18	4	121	0.80	0.97	NR
Tang 2023 [24]	RRD	146/735	134	12	8	727	0.92	0.99	0.988
Cui 2023 [25]	Mixed-type RD	6/6216	3	3	31	6185	0.50	1.00	0.910
Wang 2023 [14]	RRD	65/535	57	8	7	528	0.88	0.99	NR
Christ 2024 [27]	Mixed-type RD	176/305	153	23	14	291	0.87	0.95	0.972

Study-level diagnostic performance of deep learning models for detecting retinal detachment. “Test set (RD/non-RD)” indicates the number of retinal detachment and non-retinal detachment images (or eyes) in the primary test set selected for each study. Sensitivity was calculated as TP/(TP + FN) and specificity as TN/(TN + FP). AUC values are those reported by the original authors for the corresponding binary RD versus non-RD task; NR indicates that AUC was not reported. For studies that did not report TP, FN, FP, and TN, these values were back-calculated from the reported sensitivity, specificity, and test-set sizes. “Mixed-type RD” indicates cohorts in which the RD group included RRD and other RD subtypes; cohorts restricted to recurrent/postoperative RD were treated as an extreme-spectrum population

Abbreviations: RD = retinal detachment; RRD = rhegmatogenous retinal detachment; TP = true positive; FN = false negative; FP = false positive; TN = true negative; AUC = area under the receiver operating characteristic curve; NR = not reported

### Synthesis of results

The pooled sensitivity and specificity of DL algorithms using UWF imaging for diagnosing RD were 0.95 (95% CI: 0.94–0.96) and 0.99 (95% CI: 0.99–0.99), respectively. The forest plots for sensitivity and specificity are shown in Fig. 3. Substantial heterogeneity was observed for both sensitivity (I² = 92.4%) and specificity (I² = 90.0%).

**Fig. 4:**
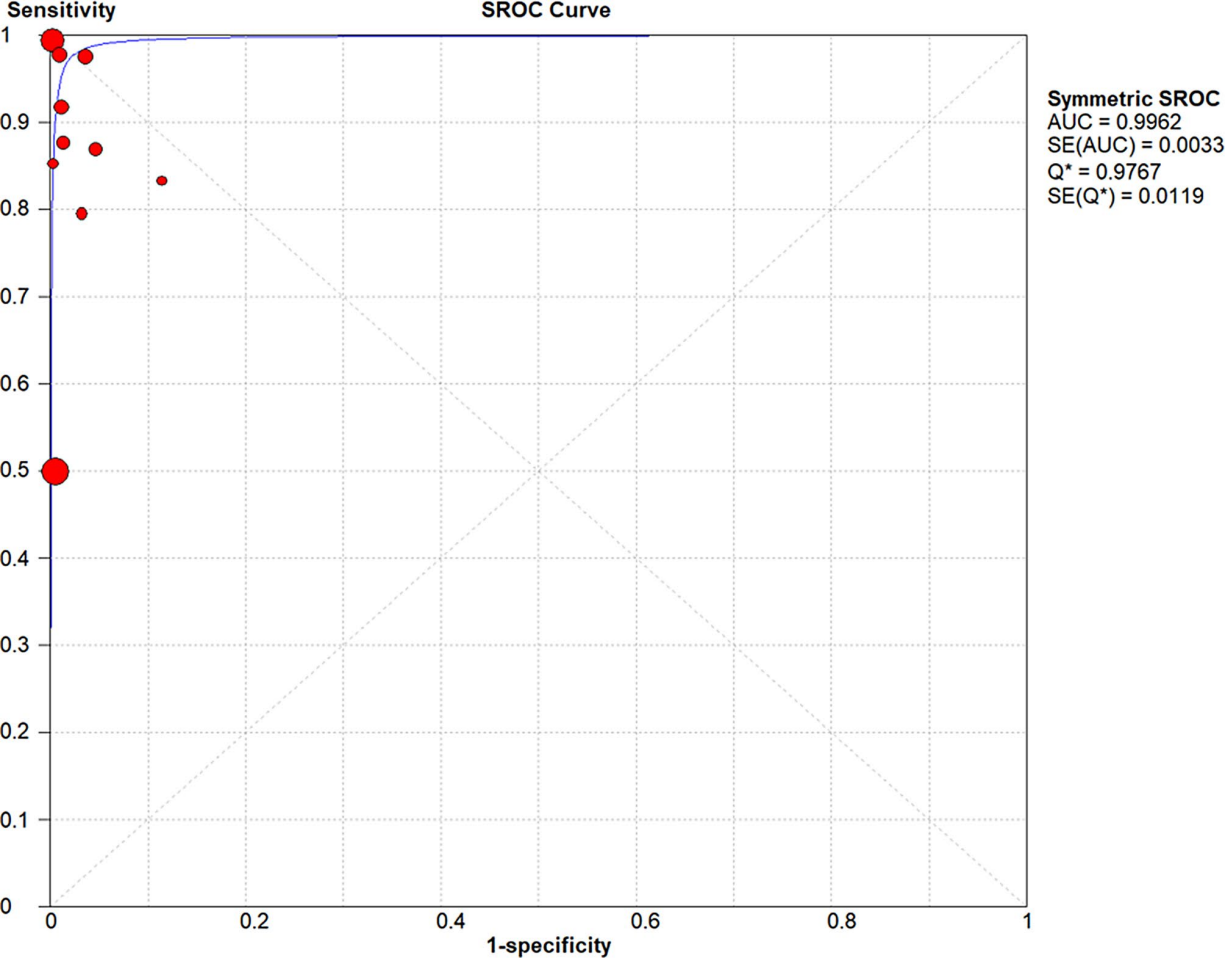
Summary receiver operating characteristic (SROC) curve for DL-based diagnosis of retinal detachment using UWF imaging. The SROC curve summarizes the overall diagnostic performance of deep learning algorithms applied to ultra-widefield fundus imaging for detecting retinal detachment. The AUC was 0.9962, indicating excellent diagnostic accuracy. Abbreviations: SROC = summary receiver operating characteristic; AUC = area under the curve; SE = standard error; Q* = Q-point on the SROC curve

The SROC curve was generated to illustrate the overall diagnostic accuracy of DL algorithms applied to ultra-widefield fundus imaging for retinal detachment detection (Fig. 4). The AUC was 0.9962, indicating excellent diagnostic performance.

### Subgroup analyses

To explore the sources of heterogeneity, we performed pre-specified subgroup analyses by dataset origin (internal vs. external) and RD subtype (Table 3). Compared with internal evaluations, external studies showed higher pooled sensitivity (0.97 [0.96–0.98] vs. 0.92 [0.90–0.94]) and similar specificity (0.99 [0.99–1.00] vs. 0.98 [0.97–0.98]); SROC AUCs remained ≈ 0.99 in both subgroups. Heterogeneity remained substantial but was slightly lower for specificity in the internal subgroup (I² 82.2%) than overall (I² 90.0%).

**Table 3:** Pooled diagnostic performance of deep learning using ultra-widefield fundus imaging for retinal detachment across key subgroups

Subgroup	k	Sensitivity (95% CI)	I² (Sens)	Specificity (95% CI)	I² (Spec)	SROC AUC
Overall	11	0.95 (0.94–0.96)	92.4%	0.99 (0.99–0.99)	90.0%	0.9962
Internal only	5	0.92 (0.90–0.94)	90.5%	0.98 (0.97–0.98)	82.2%	0.9877
External only	6	0.97 (0.96–0.98)	92.2%	0.99 (0.99–1.00)	84.1%	0.9983
RRD-only	3	0.95 (0.93–0.97)	86.6%	0.98 (0.97–0.99)	77.9%	0.9942
Mixed-type RD	7	0.95 (0.94–0.97)	94.3%	0.99 (0.99–1.00)	89.1%	0.9987
High risk-of-bias excluded	7	0.93 (0.91–0.94)	86.6%	0.99 (0.98–0.99)	81.6%	0.9933
Extreme-spectrum excluded	9	0.95 (0.94–0.96)	92.7%	0.99 (0.99–0.99)	89.3%	0.9978
High risk-of-bias + Extreme-spectrum excluded	6	0.93 (0.91–0.95)	87.2%	0.99 (0.98–0.99)	77.8%	0.9976

Summary of pooled sensitivity, specificity, heterogeneity (I²), and area under the symmetric summary receiver operating characteristic (SROC AUC) from random-effects models. Subgroup analyses explored heterogeneity by dataset origin (internal vs. external) and RD subtype (RRD-only vs. mixed-type RD). External only includes studies using independent-center and/or temporally independent test sets. Mixed-type RD indicates cohorts in which the RD group included RRD and other RD subtypes; cohorts restricted to recurrent/postoperative RD were treated as an extreme-spectrum population and evaluated in sensitivity analyses. All analyses were conducted using Meta-DiSc with random-effects pooling

Abbreviations: RD = retinal detachment; RRD = rhegmatogenous retinal detachment; AUC = area under the curve; SROC = summary receiver-operating characteristic

When restricting to RRD-only studies (k = 3), pooled estimates were 0.95 (0.93–0.97) for sensitivity and 0.98 (0.97–0.99) for specificity (AUC 0.9942), with modestly lower I² (86.6% and 77.9%, respectively) than overall. In the clinically relevant subset of mixed-type RD cohorts (k = 7), performance was essentially unchanged from the overall analysis (sensitivity 0.95 [0.94–0.97]; specificity 0.99 [0.99–1.00]; AUC 0.9987), while heterogeneity remained high (I² 94.3% and 89.1%).

Sensitivity analyses excluding high-risk-of-bias studies (Ohsugi 2017 [26], Cao 2022 [22], Antaki 2023 [13], and Cui 2023 [25]) and/or extreme-spectrum cohorts (population screening and recurrent/postoperative RD; Cui 2023 [25] and Zhou 2022 [21]) yielded similar pooled estimates (sensitivity 0.93–0.95; specificity 0.99; SROC AUC 0.9933–0.9978), supporting the robustness of the primary findings. Heterogeneity remained substantial across these sensitivity analyses (Table 3; Supplementary Figure S3).

### Reporting bias

For the overall synthesis (k = 11), Deeks’ funnel asymmetry test—weighted regression of log diagnostic odds ratio on 1/√effective sample size—showed no evidence of small-study effects (slope 6.53, *p* = 0.27). Because subgroup syntheses contained fewer than 10 studies and heterogeneity was high, reporting bias was not assessed for subgroups; accordingly, the risk due to missing results remains unclear.

## Discussion

This meta-analysis indicates that DL applied to UWF imaging achieves high diagnostic accuracy for RD, albeit with substantial heterogeneity across studies. To our knowledge, it is the first meta-analysis to quantitatively evaluate DL for RD detection on UWF images; prior scoping reviews summarized UWF DL applications but did not report pooled diagnostic accuracy for RD [10, 28].

DL algorithms for other ocular diseases, such as diabetic retinopathy and age-related macular degeneration, have also shown high diagnostic accuracy. For example, a recent systematic review on diabetic retinopathy reported sensitivities ≥ 85% with specificities often below 80% [29], while a meta-analysis on age-related macular degeneration found a pooled sensitivity of 0.94, specificity of 0.97, and an AUC of 0.9925 [30]. The performance observed in this study for RD is comparable to, or in some contexts higher than, that reported for these conditions, supporting its potential clinical utility.

Most studies were retrospective, and two were prospective (Cui et al., Zhou et al.) [21, 25]. Sample sizes and case spectra varied widely, and reported AUCs ranged from 0.910 to 1.000. Near-ceiling performance appeared in Li 2020, Zhang 2021, and Sun 2023 (AUC 1.000, 1.000, and 0.998) [19, 20, 23]. In contrast, performance was lower in postoperative-only cohorts or in screening settings with extreme class imbalance [21, 25], and one AutoML multiclass study reported a high yet comparatively lower area under the precision–recall curve (AUPRC) (0.946) [13]. Across studies, devices (Optos 200Tx/Daytona/P200; Clarus 500), task formulations (binary, multilabel, detection), and unspecified decision thresholds likely contributed to between-study heterogeneity (Table 1; Supplementary Table S2).

Across the included studies, DL implementations varied in architecture and task formulation. Most models were CNN-based (Inception family, EfficientNet, seResNeXt, Inception-ResNet-V2), with some using object detection (YOLOX) or code-free AutoML [13]. Several studies adopted multilabel frameworks that modeled RD together with peripheral lesions such as retinal tears and lattice degeneration [20, 22]. Sun et al. achieved clinician-level accuracy with an EfficientNet multilabel model while maintaining efficient inference [23]. Cao et al. reported high per-class RD performance on external cohorts in a multiclass system, although AUROC for RD was not applicable in that setting [22]. These architectural and task differences likely contributed to between-study variability; model and device details are summarized in Supplementary Table S2.

There was considerable variation in the reference standards used across studies. While most studies relied on diagnoses made by retinal specialists, some failed to specify the diagnostic criteria in detail. The inclusion of both studies that employed consensus diagnoses by multiple specialists and those that may have relied on individual diagnoses could have contributed to the variability in the results.

Substantial heterogeneity persisted for both sensitivity (I² = 92.4%) and specificity (I² = 90.0%). In dataset-origin subgroups, external evaluations showed higher pooled sensitivity than internal (0.97 vs. 0.92) with similarly high specificity and SROC AUCs ≈ 0.99, indicating that part of the variability reflects dataset origin/generalizability rather than inconsistent accuracy (Table 3). Aligning RD subtype modestly reduced heterogeneity in the RRD-only subset (I² 86.6% for sensitivity; 77.9% for specificity), whereas the mixed-type RD subset was essentially unchanged from overall, suggesting that case spectrum also contributes (Table 3). Taken together with differences in imaging device (Optos and Clarus), DL architecture (Inception family, EfficientNet, seResNeXt, detection models and AutoML), and the reporting of reference standards, we cannot determine from the present synthesis whether heterogeneity primarily reflects structural factors (population spectrum, device and task formulation) or random variation. More standardized reporting of dataset origin, control definitions, decision thresholds and external validation would facilitate clearer attribution in future studies.

This meta-analysis showed that combining UWF fundus imaging with DL algorithms provides high diagnostic accuracy for detecting RD, with pooled sensitivity and specificity of 0.95 and 0.99, respectively. Although identifying a typical RD is usually straightforward for experienced ophthalmologists using indirect ophthalmoscopy or B-scan ultrasonography, these modalities are examiner dependent and not always readily available in primary or emergency-care settings. Traditional techniques like indirect ophthalmoscopy and B-scan ultrasonography are limited by examiner dependency and restricted peripheral visualization. In contrast, DL applied to UWF images has the potential to facilitate wide-field, noninvasive, and standardized retinal assessment, particularly when UWF images are acquired by non-specialists and interpreted remotely. DL-assisted systems may also reduce clinician workload and help address disparities in diagnostic access, especially in underserved regions. In practice, such systems are most likely to be useful as triage or “second-reader” tools by flagging suspected RD on UWF images obtained in high-volume clinics, emergency departments, or community-based screening programs, so that patients who require urgent specialist assessment can be prioritized. They could serve as first-line screening tools to detect vision-threatening RD earlier, potentially improving outcomes and optimizing resource use.

Although the pooled sensitivity, specificity, and AUC suggest excellent overall diagnostic accuracy, the substantial heterogeneity means that these pooled values should be interpreted with caution. In particular, AUCs were lower in more challenging, context-specific settings such as rural population screening and postoperative recurrent RD cohorts (Cui [25] and Zhou [21]; AUC 0.91 and 0.93), where pronounced class imbalance and distinct case spectra also coincided with modestly reduced sensitivity. Nevertheless, sensitivity analyses excluding high-risk-of-bias studies and/or these extreme-spectrum cohorts yielded similar pooled estimates, suggesting that the overall findings were robust to these exclusions despite persistent heterogeneity (Table 3; Supplementary Figure S3). Consistent with previous reports on DL models, these findings also suggest that performance estimates derived from small, enriched internal test sets may be optimistic, and that external evaluations provide a more realistic indication of generalizability. These findings suggest that current DL models may be more reliable as adjunctive tools in hospital-based UWF RD cohorts with case spectra and imaging protocols similar to the development data, whereas extrapolation of performance estimates to settings such as community-based screening programs or highly imbalanced, specialized settings should be made with caution. At the same time, the slightly higher pooled sensitivity observed in the external subgroup compared with the internal subgroup should be interpreted cautiously, because this subgroup comprises a small number of studies, including context-specific external cohorts such as population-based screening and postoperative recurrent RD, and the apparent difference is more likely to reflect differences in case mix, class balance, and operating thresholds than a true superiority of externally evaluated models.

This meta-analysis has several limitations. First, substantial heterogeneity remained for both sensitivity and specificity, and non-RD controls varied from healthy eyes to eyes with other retinal conditions (Supplementary Table S2), which likely affected specificity and contributed to this heterogeneity. Our prespecified subgroup analyses (internal vs. external datasets; RD subtype) suggest that variability was partly structural, reflecting dataset origin/generalizability and case spectrum, while residual differences likely arose from imaging devices (Optos vs. Clarus), DL architectures (including classification vs. detection/AutoML), and inconsistencies in reference standards. Second, some studies did not clearly report diagnostic criteria or grader procedures, which may affect reliability. Third, sample sizes varied widely (89–6,222 images), potentially affecting the stability of pooled estimates; therefore, generalization should be cautious. Process-wise, our search covered two major databases plus hand-searching, but studies indexed elsewhere or in the grey literature may have been missed. Subgroup analyses were intentionally limited to a small set of prespecified contrasts and were likely underpowered; meta-regression was not performed for the same reason.

In conclusion, this systematic review and meta-analysis demonstrated that DL algorithms applied to ultra-widefield fundus imaging achieve high diagnostic accuracy for detecting RD, with pooled sensitivity and specificity of 0.95 and 0.99, respectively, and an AUC of 0.9962. Despite notable heterogeneity among studies due to differences in DL architectures, imaging devices, and reference standards, the generally high performance suggests that DL combined with UWF imaging is likely to serve as a valuable adjunctive tool in RD diagnosis.

## Supplementary Information

Below is the link to the electronic supplementary material.

Supplementary Material 1

Supplementary Material 2

Supplementary Material 3

Supplementary Material 4

## Data Availability

All data analyzed in this study are derived from previously published articles and are included in the present manuscript and supplementary materials. No new data were generated.

## Abbreviations

AUC: Area under the curve
AutoML: Automated machine learning
CI: Confidence interval
CNN: Convolutional neural network
DOR: Diagnostic odds ratio
DL: Deep learning
DR: Diabetic retinopathy
ESS: Effective sample size
FN: False negative
FP: False positive
I²: Inconsistency index (I-squared)
Inception-ResNet-V2: Inception residual network v2
PRISMA-DTA: Preferred Reporting Items for Systematic Reviews and Meta-Analyses of Diagnostic Test Accuracy
PROSPERO: International Prospective Register of Systematic Reviews
QUADAS-2: Quality Assessment of Diagnostic Accuracy Studies-2
RD: Retinal detachment
RRD: Rhegmatogenous retinal detachment
ROC: Receiver operating characteristic
SE: Standard error
SROC: Summary receiver operating characteristic
TN: True negative
TP: True positive
UWF: Ultra-widefield (fundus imaging)
UMIN-CTR: University Hospital Medical Information Network Clinical Trials Registry
YOLOX: You Only Look Once, version X (object-detection network)
EfficientNet-B0/-B7: EfficientNet architectures (versions B0 and B7)
seResNeXt: Squeeze-and-excitation ResNeXt
Q*: Q‑point on the SROC curve (point where sensitivity equals specificity)
AUPRC: Area under the precision–recall curve
