# Supplementary material for: Diagnostic accuracy of deep learning using ultra-widefield fundus imaging for retinal detachment: a systematic review and meta-analysis
Source: BMC Ophthalmol. 2026 Jan 3;26:60. doi: 10.1186/s12886-025-04605-8 (PMC12866021; doi:10.1186/s12886-025-04605-8)
Supplement: Supplementary file 1 — Supplementary Material 1 [file 12886_2025_4605_MOESM1_ESM.pdf]

Supplementary Material – Search Strategies

Database Search Strategies

A comprehensive search was performed in PubMed and Web of Science to identify eligible studies. The search strategy combined terms related to deep learning, retinal detachment, and ultra-widefield imaging. No restrictions on language, publication date, or document type were applied. The last search for both databases was conducted on May 22, 2025.

PubMed Search Strategy

(last searched May 22, 2025)

Number of records retrieved: 115

("deep learning" OR AI OR "artificial intelligence" OR Convolutional OR convolution OR CNN OR Attention OR "deep learning systems" OR DLSs OR DLS OR "machine learning" OR "neural network")

AND

("retinal detachment" OR "retina detachment" OR "retinal tear" OR "retina tear" OR "retinal break" OR "retina break" OR "rhegmatogenous retinal detachment")

AND

("ultra-widefield" OR "ultrawide field" OR "ultrawidefield" OR "ultra wide field" OR "ultra-wide-field" OR "wide-field fundus" OR "wide field fundus" OR "widefield fundus" OR "wide-field-fundus" OR widefieldfundus OR "wide-angle fundus" OR "wide-angle-fundus" OR "wide angle fundus" OR wideanglefundus OR "widefield imaging" OR "wide-field imaging" OR "wide field imaging" OR "wide-field-imaging" OR widefieldimaging OR "200-degree" OR "200-degree retinal" OR "panretinal" OR UWF OR "UWF imaging" OR Optos OR California OR Daytona OR Clarus OR Mirante OR Spectralis)

Web of Science Search Strategy

(last searched May 22, 2025)

Number of records retrieved: 36

("deep learning" OR AI OR "artificial intelligence" OR Convolutional OR convolution OR CNN OR Attention OR "deep learning systems" OR DLSs OR DLS OR "machine learning" OR "neural network")

AND

("retinal detachment" OR "retina detachment" OR "retinal tear" OR "retina tear" OR "retinal break" OR "retina break" OR "rhegmatogenous retinal detachment")

AND

("ultra-widefield" OR "ultrawide field" OR "ultrawidefield" OR "ultra wide field" OR "ultra-wide-field" OR "wide-field fundus" OR "wide field fundus" OR "widefield fundus" OR "wide-field-fundus" OR widefieldfundus OR "wide-angle fundus" OR "wide-angle-fundus" OR "wide angle fundus" OR wideanglefundus OR "widefield imaging" OR "wide-field imaging" OR "wide field imaging" OR "wide-field-imaging" OR widefieldimaging OR "200-degree" OR "200-degree retinal" OR "panretinal" OR UWF OR "UWF imaging" OR Optos OR California OR Daytona OR Clarus OR Mirante OR Spectralis)
