# Supplementary material for: Diagnostic accuracy of deep learning using ultra-widefield fundus imaging for retinal detachment: a systematic review and meta-analysis
Source: BMC Ophthalmol. 2026 Jan 3;26:60. doi: 10.1186/s12886-025-04605-8 (PMC12866021; doi:10.1186/s12886-025-04605-8)
Supplement: Supplementary file 2 — Supplementary Material 2 [file 12886_2025_4605_MOESM2_ESM.pdf]

Supplementary Table S1. Excluded studies that appeared eligible and reasons

Author, year	Reason for exclusion
Li, 2019	Peripheral lesions (lattice degeneration/retinal breaks); not RD diagnosis.
Ahmad, 2020	Educational/review piece; not original research.
Zhang, 2021 (DeepUWF)	Mixed endpoint (retinal tear & RD) with no extractable 2×2 data for RD alone.
Bhambra, 2022	Scoping review; not an original diagnostic accuracy study.
Oh, 2022	Retinal break detection/localization (YOLO); not RD diagnosis meta-analyzable for our outcomes.
Catania, 2023	Conference abstract; outcome was late recurrence prediction after RD surgery, not initial RD diagnosis.
Tang, 2024	Review article; not an original diagnostic accuracy study.
Li, 2024	Weakly supervised localization/management of RD regions; not a diagnostic accuracy study of RD detection suitable for pooling.
Miao, 2024	Compared USG/UWF/IDO for retinal breaks; not DL-based RD diagnosis.
Catania, 2024	Prediction of late recurrence after RD surgery; outcome not initial RD diagnosis.
Tabuchi, 2024 (Real-World Performance)	Not deep learning–based index test for RD on UWF images.
Tabuchi, 2024 (Synthetic images training)	Educational intervention using synthetic images; not a diagnostic accuracy study.
Gan, 2025	PVR identification/severity/risk using multimodal imaging; not UWF DL diagnosis of RD.

Note: Details correspond to the studies screened at the full-text stage but excluded after eligibility assessment (see Figure 1, PRISMA flow diagram).

Abbreviations: RD, retinal detachment; DL, deep learning; UWF, ultra-widefield; USG, ultrasonography; IDO, indirect ophthalmoscopy; PVR, proliferative vitreoretinopathy; YOLO, You Only Look Once.

Supplementary References

1. Li, Z. *et al.* A deep learning system for identifying lattice degeneration and retinal breaks using ultra-widefield fundus images. *Ann. Transl. Med.* 7, 618 (2019).
2. Ahmad, B.U., Kim, J.E. & Rahimy, E. Fundamentals of artificial intelligence for ophthalmologists. *Curr. Opin. Ophthalmol.* **31**, 303–311 (2020).
3. Zhang, W., Zhao, X., Chen, Y., Zhong, J. & Yi, Z. DeepUWF: An automated ultra-wide-field fundus screening system via deep learning. *IEEE J. Biomed. Health Inform.* **25**, 2988–2996 (2021).
4. Bhambra, N., Antaki, F., Malt, F.E., Xu, A. & Duval, R. Deep learning for ultra-widefield imaging: a scoping review. *Graefes Arch. Clin. Exp. Ophthalmol.* **260**, 3737–3778 (2022).
5. Oh, R., Oh, B.L., Lee, E.K., Park, U.C., Yu, H.G. & Yoon, C.K. Detection and localization of retinal breaks in ultrawidefield fundus photography using a YOLO v3 architecture-based deep learning model. Retina 42, 1889–1896 (2022).
6. Catania, F. *et al.* Deep learning for prediction of rhegmatogenous retinal detachment relapse using preoperative and postoperative wide field imaging. *Invest. Ophthalmol. Vis. Sci.* **64**, 3 (2023).
7. Tang, Q.Q., Yang, X.G., Wang, H.Q., Wu, D.W. & Zhang, M.X. Applications of deep learning for detecting ophthalmic diseases with ultrawide-field fundus images. *Int. J. Ophthalmol.* **17**, 188–200 (2024).
8. Li, H., Cao, J., You, K., Zhang, Y. & Ye, J. Artificial intelligence-assisted management of retinal detachment from ultra-widefield fundus images based on weakly-supervised approach. *Front. Med.* **11**, 1326004 (2024).
9. Miao, A. *et al.* Comparison of B-scan ultrasonography, ultra-widefield fundus imaging, and indirect ophthalmoscopy in detecting retinal breaks in cataractous eyes. *Eye* **38**, 2619–2624 (2024).
10. Catania, F. *et al.* Deep learning for prediction of late recurrence of retinal detachment using preoperative and postoperative ultra-wide field imaging. *Acta Ophthalmol.* **102**, e984–e993 (2024).
11. Tabuchi, H., Engelmann, J., Deguchi, H., Ishitobi, N. & Bernabeu, M. Real-world performance evaluation of an AI model for detecting retinal detachment in UWF fundus images. *Invest. Ophthalmol. Vis. Sci.* **65**, 2 (2024).
12. Tabuchi, H. *et al.* Using artificial intelligence to improve human performance: efficient retinal disease detection training with synthetic images. *Br. J. Ophthalmol.* **108**, 1430–1435 (2024).
13. Gan, F. *et al.* Development and validation of the Artificial Intelligence-Proliferative Vitreoretinopathy (AI-PVR) Insight system for deep learning-based diagnosis and postoperative risk prediction in proliferative vitreoretinopathy using multimodal fundus imaging. *Quant. Imaging Med. Surg.* **15**, 2774–2788 (2025).
