# Supplementary material for: Diagnostic accuracy of deep learning using ultra-widefield fundus imaging for retinal detachment: a systematic review and meta-analysis
Source: BMC Ophthalmol. 2026 Jan 3;26:60. doi: 10.1186/s12886-025-04605-8 (PMC12866021; doi:10.1186/s12886-025-04605-8)
Supplement: Supplementary file 3 — Supplementary Material 3 [file 12886_2025_4605_MOESM3_ESM.pdf]

Supplementary Table S2. Detailed characteristics of included studies and deep learning model information.

Study	Reference standard	Imaging device	Deep learning architecture	Controls (non-RD group)	Cut-off	Dataset used for evaluation
Ohsugi 2017 [26]	Unclear	Optos (200Tx)	CNN	Normal only	Not reported	Internal dataset
Li 2020 [19]	Unclear	Not specified	Not specified	Normal + other retinal diseases	Not reported	External dataset from the Zhongshan Ophthalmic Centre
Zhang 2021 [20]	3 retinal specialists	Optos (Daytona/PLC)	CNN seResNext50	Normal peripheral retina + other target lesions	Not reported	Internal dataset
Zhou 2022 [21]	2 experienced retinal specialists	Clarus 500	Inception-ResNet-V2	Post-operative reattached eyes	Not reported	Independent dataset (different time period)
Cao 2022 [22]	Team of 5 ophthalmologists (2 junior, 2 senior, 1 specialist)	Optos (Daytona)	ResNeXt-50 + CAFPN + AdaBoost-SVM	Normal + other diseases	Argmax (multiclass)	External dataset from the First Affiliated Hospital of University of Science and Technology
Sun 2023 [23]	3 ophthalmologists	Optos (Daytona / 200Tx)	EfficientNet-B7	Normal + other diseases	Argmax (multiclass)	External dataset from Wuhan Optics Valley Central Hospital
Antaki 2023 [13]	Unclear	Optos (200Tx)	AutoML	Normal + RVO + RP	Argmax (multiclass)	Internal dataset
Tang 2023 [24]	2 trained retinal ophthalmologists	Optos (Daytona / P200)	Inception	Normal + peripheral lesions	Not reported	Internal dataset
Cui 2023 [25]	Unclear	Optos (Daytona / P200T)	Unclear (previous DLS by Li 2020)	General screening non-RD	Not reported	External dataset from patients in rural areas

Wang 2023 [14]	5 retinal specialists	Optos (Daytona)	YOLOX	Normal + peripheral lesions	IoU > 0.5	External dataset from West China Hospital
Christ 2024 [27]	2 retinal specialists	Optos (PLC)	EfficientNet-b0	No-study-lesion + retinal break only	Argmax (multiclass)	Internal dataset

Cut-off: Probability or decision threshold used to define positive classification. “Argmax (multiclass)” = highest-probability class was selected as output. “IoU” = intersection-over-union threshold for object detection.

Dataset: “Internal” = same institution and dataset; “External” = independent cohort; “Temporal-external” = same institution but different time period.

Abbreviations:

AdaBoost-SVM, Adaptive Boosting–Support Vector Machine; AUC, area under the curve; CAFPN, Channel Attention Feature Pyramid Network; CNN, convolutional neural network; DLS, deep learning system; DL, deep learning; IoU, intersection over union; RD, retinal detachment; RRD, rhegmatogenous retinal detachment; RVO, retinal vein occlusion; RP, retinitis pigmentosa; SROC, summary receiver operating characteristic; UWF, ultra-widefield (fundus imaging); YOLOX, “You Only Look Once”, version X (object-detection network); ResNeXt-50, residual network with aggregated transformations (50 layers); EfficientNet-B0/B7, EfficientNet architectures (versions B0 and B7); Inception-ResNet-V2, Inception residual network v2; Optos (200Tx, Daytona, P200, PLC), ultra-widefield fundus cameras by Optos PLC; Clarus 500, widefield fundus camera by Carl Zeiss Meditec.
