# Supplementary material for: Diagnostic accuracy of deep learning using ultra-widefield fundus imaging for retinal detachment: a systematic review and meta-analysis
Source: BMC Ophthalmol. 2026 Jan 3;26:60. doi: 10.1186/s12886-025-04605-8 (PMC12866021; doi:10.1186/s12886-025-04605-8)
Supplement: Supplementary file 4 — Supplementary Material 4 [file 12886_2025_4605_MOESM4_ESM.pdf]

Supplementary Figure S3. Sensitivity analysis excluding high-risk-of-bias and extreme-spectrum studies for deep learning–based retinal detachment detection using ultra-widefield imaging.

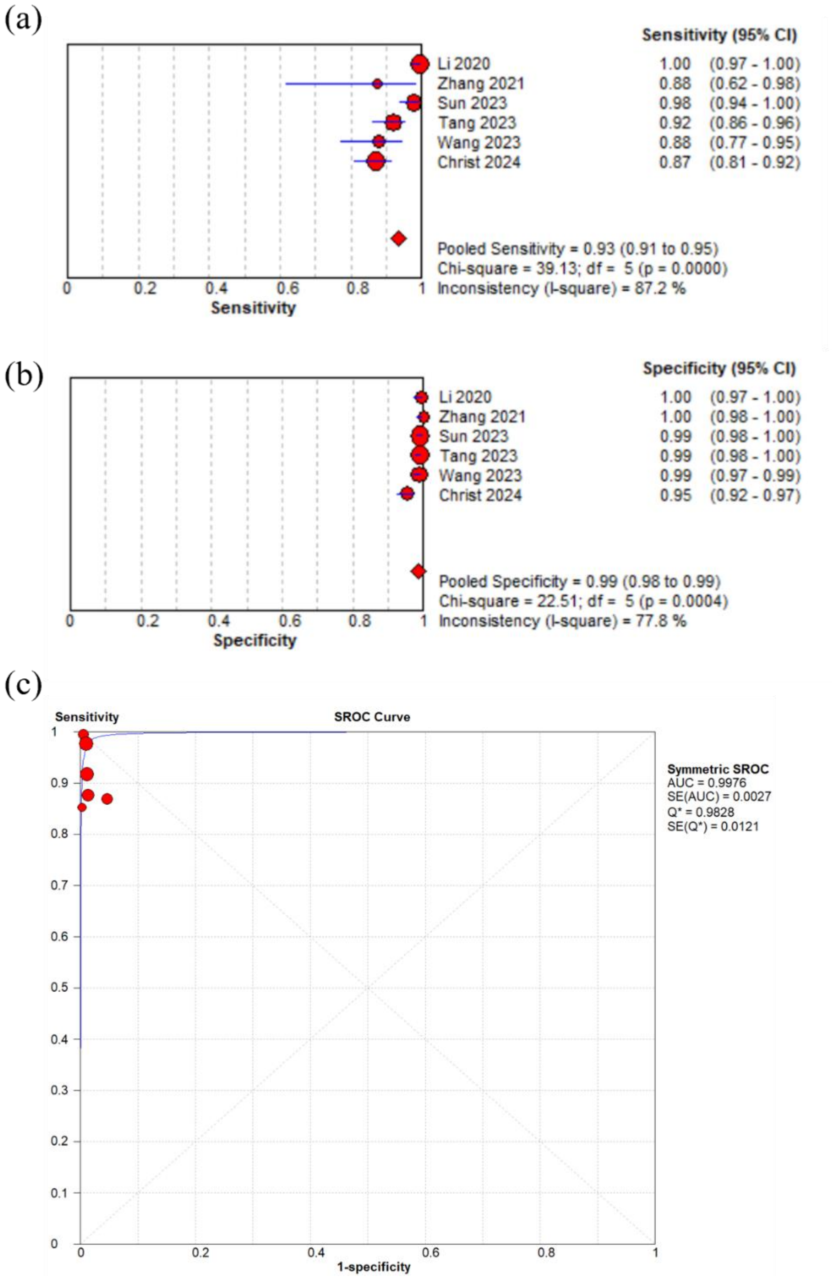

(a) Forest plot of sensitivity.

(b) Forest plot of specificity.

(c) Symmetric summary receiver operating characteristic (SROC) curve and area under the curve (AUC).

This sensitivity analysis excludes studies at high risk of bias and extreme-spectrum populations (population screening and recurrent/postoperative retinal detachment cohorts). Pooled sensitivity and specificity were estimated using univariate random-effects models (DerSimonian–Laird) in Meta-DiSc v1.4 with a continuity correction of 0.5 when required. The SROC curve and AUC were derived using the Moses–Littenberg method. Abbreviations: AUC, area under the curve; CI, confidence interval; I^2, inconsistency (I-squared); SE, standard error; SROC, summary receiver operating characteristic; Q*, Q-point on the SROC curve (point where sensitivity equals specificity).
